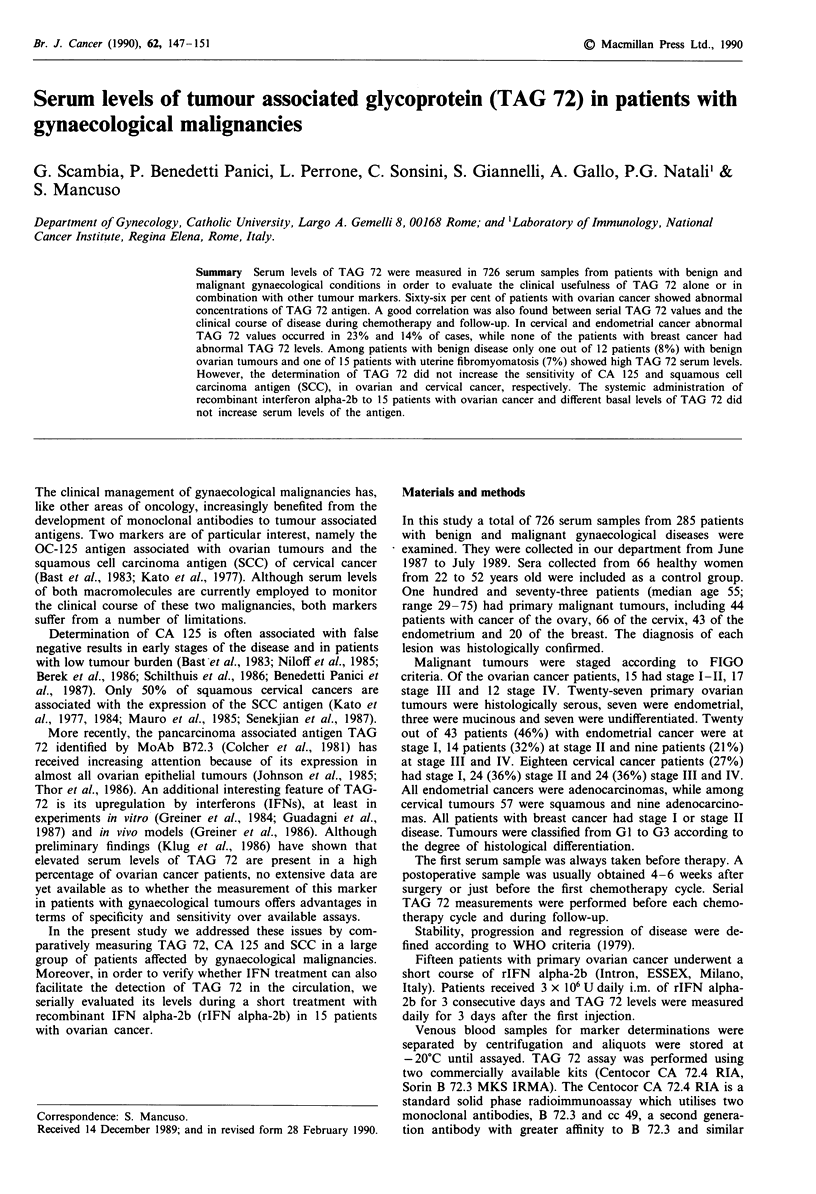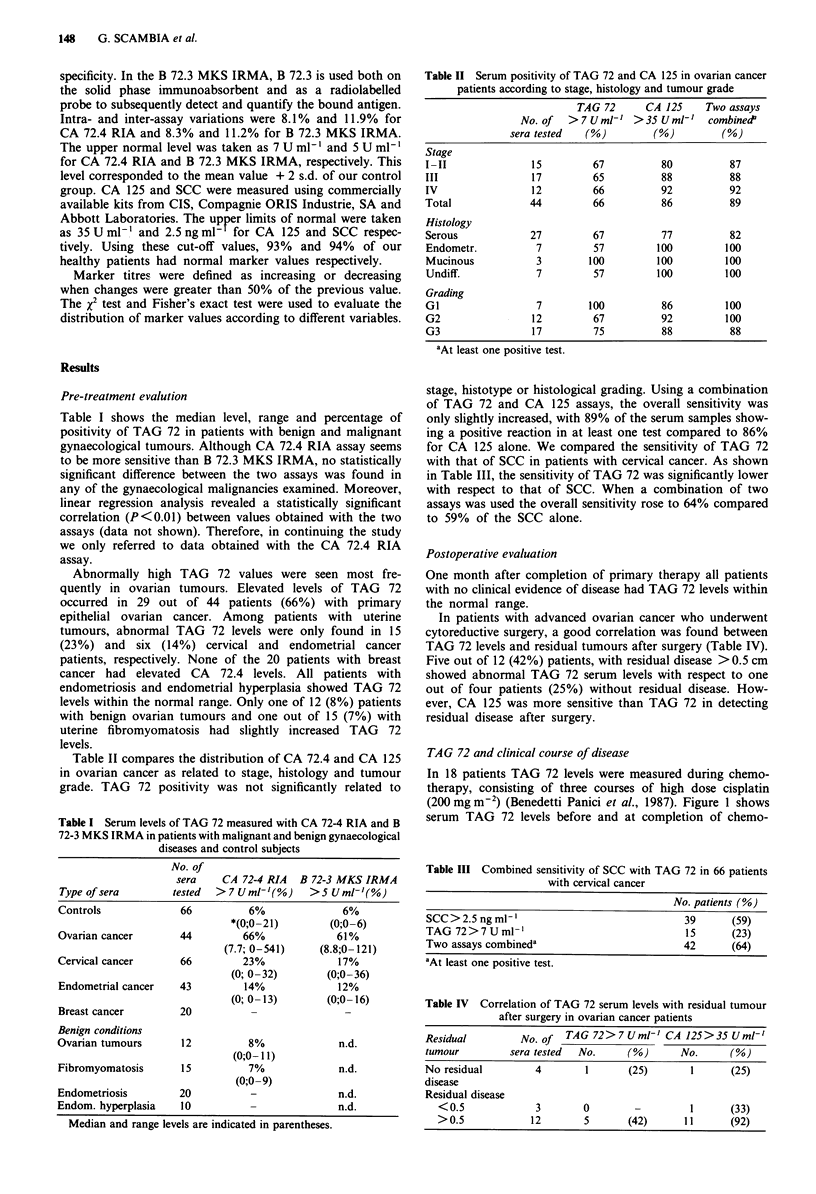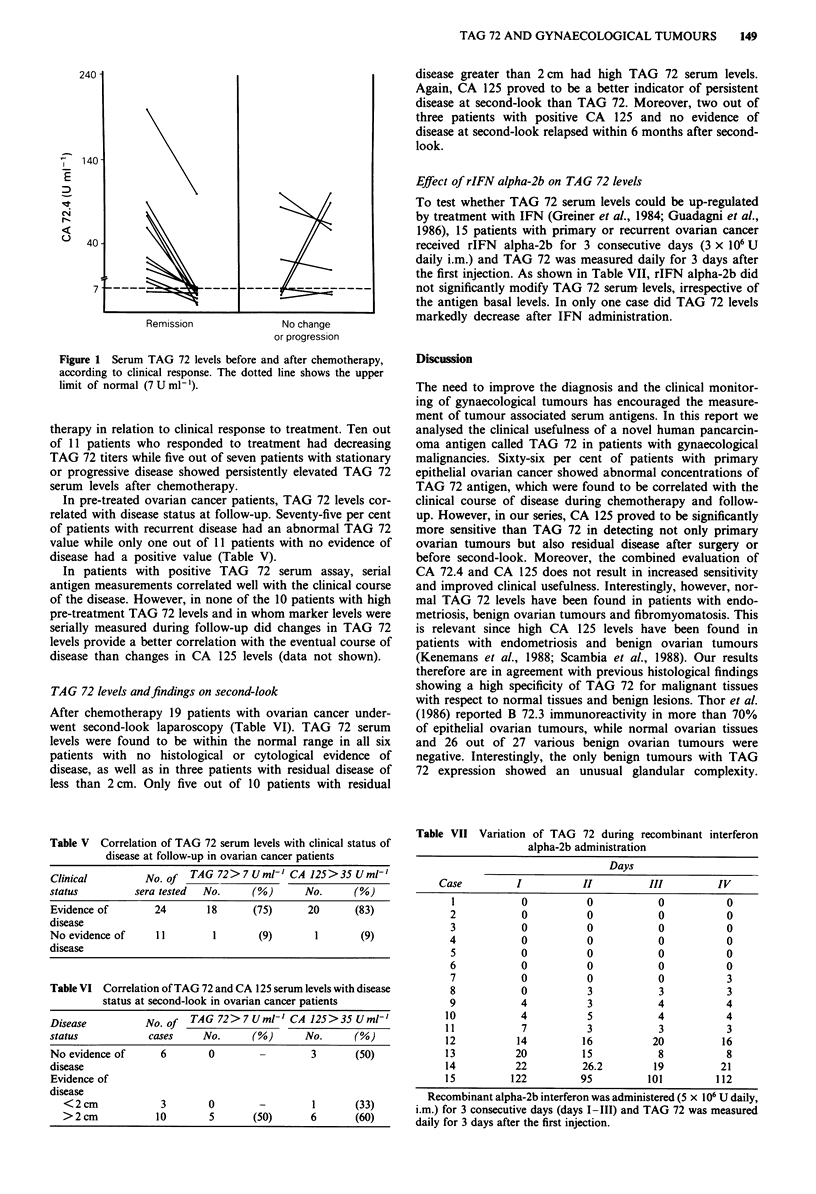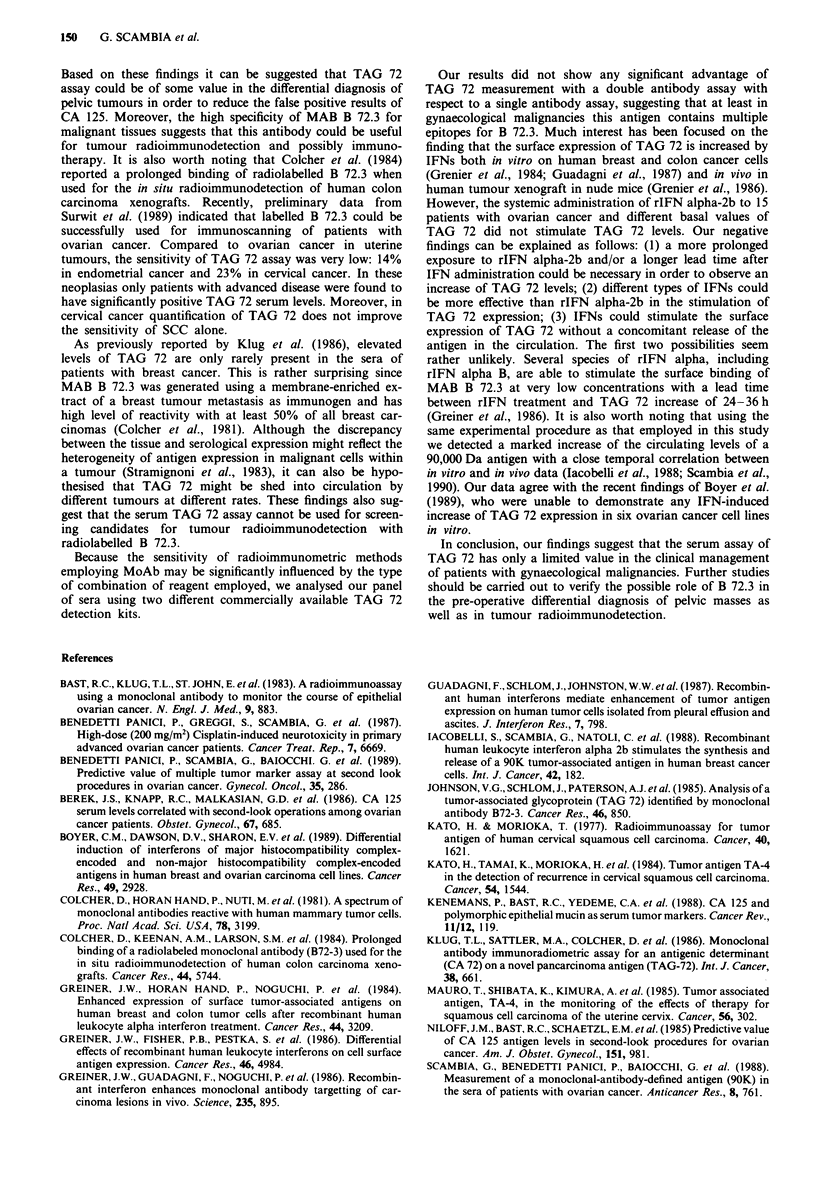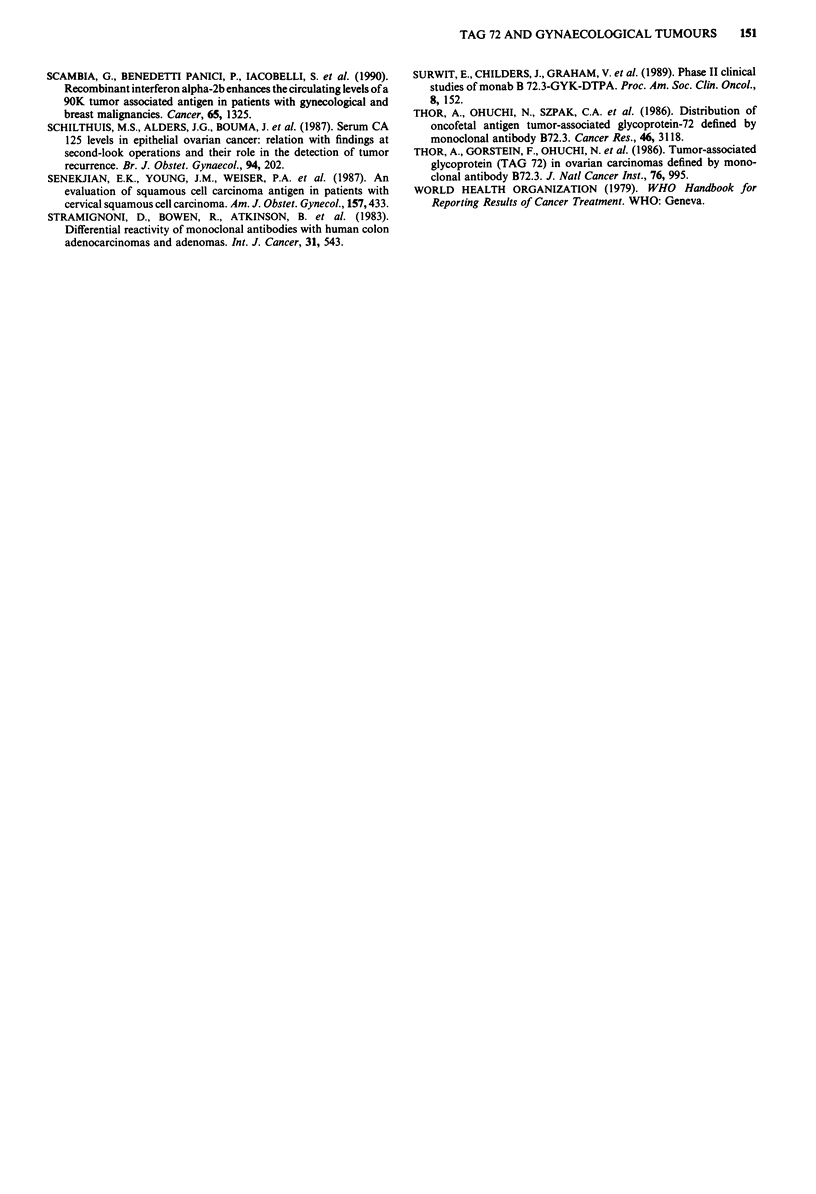# Serum levels of tumour associated glycoprotein (TAG 72) in patients with gynaecological malignancies.

**DOI:** 10.1038/bjc.1990.248

**Published:** 1990-07

**Authors:** G. Scambia, P. Benedetti Panici, L. Perrone, C. Sonsini, S. Giannelli, A. Gallo, P. G. Natali, S. Mancuso

**Affiliations:** Department of Gynecology, Catholic University, Rome, Italy.

## Abstract

Serum levels of TAG 72 were measured in 726 serum samples from patients with benign and malignant gynaecological conditions in order to evaluate the clinical usefulness of TAG 72 alone or in combination with other tumour markers. Sixty-six per cent of patients with ovarian cancer showed abnormal concentrations of TAG 72 antigen. A good correlation was also found between serial TAG 72 values and the clinical course of disease during chemotherapy and follow-up. In cervical and endometrial cancer abnormal TAG 72 values occurred in 23% and 14% of cases, while none of the patients with breast cancer had abnormal TAG 72 levels. Among patients with benign disease only one out of 12 patients (8%) with benign ovarian tumours and one of 15 patients with uterine fibromyomatosis (7%) showed high TAG 72 serum levels. However, the determination of TAG 72 did not increase the sensitivity of CA 125 and squamous cell carcinoma antigen (SCC), in ovarian and cervical cancer, respectively. The systemic administration of recombinant interferon alpha-2b to 15 patients with ovarian cancer and different basal levels of TAG 72 did not increase serum levels of the antigen.


Br. J. Cancer (1990), 62, 147-151 © Macmillan Press Ltd., 1990

### Serum levels of tumour associated glycoprotein (TAG 72) in patients with gynaecological malignancies

G. Scambia, P. Benedetti Panici, L. Perrone, C. Sonsini, S. Giannelli, A. Gallo, P.G. Natali[1] & S. Mancuso

*Department of Gynecology, Catholic University, Largo A. Gemelli 8, 00168 Rome; and [1]Laboratory of Immunology, National Cancer Institute, Regina Elena, Rome, Italy.*

**Summary** Serum levels of TAG 72 were measured in 726 serum samples from patients with benign and malignant gynaecological conditions in order to evaluate the clinical usefulness of TAG 72 alone or in combination with other tumour markers. Sixty-six per cent of patients with ovarian cancer showed abnormal concentrations of TAG 72 antigen. A good correlation was also found between serial TAG 72 values and the clinical course of disease during chemotherapy and follow-up. In cervical and endometrial cancer abnormal TAG 72 values occurred in 23% and 14% of cases, while none of the patients with breast cancer had abnormal TAG 72 levels. Among patients with benign disease only one out of 12 patients (8%) with benign ovarian tumours and one of 15 patients with uterine fibromyomatosis (7%) showed high TAG 72 serum levels. However, the determination of TAG 72 did not increase the sensitivity of CA 125 and squamous cell carcinoma antigen (SCC), in ovarian and cervical cancer, respectively. The systemic administration of recombinant interferon alpha-2b to 15 patients with ovarian cancer and different basal levels of TAG 72 did not increase serum levels of the antigen.

The clinical management of gynaecological malignancies has, like other areas of oncology, increasingly benefited from the development of monoclonal antibodies to tumour associated antigens. Two markers are of particular interest, namely the OC-125 antigen associated with ovarian tumours and the squamous cell carcinoma antigen (SCC) of cervical cancer (Bast *et al.*, 1983; Kato *et al.*, 1977). Although serum levels of both macromolecules are currently employed to monitor the clinical course of these two malignancies, both markers suffer from a number of limitations.

Determination of CA 125 is often associated with false negative results in early stages of the disease and in patients with low tumour burden (Bast *et al.*, 1983; Niloff *et al.*, 1985; Berek *et al.*, 1986; Schilthuis *et al.*, 1986; Benedetti Panici *et al.*, 1987). Only 50% of squamous cervical cancers are associated with the expression of the SCC antigen (Kato *et al.*, 1977, 1984; Mauro *et al.*, 1985; Senekjian *et al.*, 1987).

More recently, the pancarcinoma associated antigen TAG 72 identified by MoAb B72.3 (Colcher *et al.*, 1981) has received increasing attention because of its expression in almost all ovarian epithelial tumours (Johnson *et al.*, 1985; Thor *et al.*, 1986). An additional interesting feature of TAG-72 is its upregulation by interferons (IFNs), at least in experiments *in vitro* (Greiner *et al.*, 1984; Guadagni *et al.*, 1987) and *in vivo* models (Greiner *et al.*, 1986). Although preliminary findings (Klug *et al.*, 1986) have shown that elevated serum levels of TAG 72 are present in a high percentage of ovarian cancer patients, no extensive data are yet available as to whether the measurement of this marker in patients with gynaecological tumours offers advantages in terms of specificity and sensitivity over available assays.

In the present study we addressed these issues by comparatively measuring TAG 72, CA 125 and SCC in a large group of patients affected by gynaecological malignancies. Moreover, in order to verify whether IFN treatment can also facilitate the detection of TAG 72 in the circulation, we serially evaluated its levels during a short treatment with recombinant IFN alpha-2b (rIFN alpha-2b) in 15 patients with ovarian cancer.

Correspondence: S. Mancuso.
Received 14 December 1989; and in revised form 28 February 1990.

### Materials and methods

In this study a total of 726 serum samples from 285 patients with benign and malignant gynaecological diseases were examined. They were collected in our department from June 1987 to July 1989. Sera collected from 66 healthy women from 22 to 52 years old were included as a control group. One hundred and seventy-three patients (median age 55; range 29-75) had primary malignant tumours, including 44 patients with cancer of the ovary, 66 of the cervix, 43 of the endometrium and 20 of the breast. The diagnosis of each lesion was histologically confirmed.

Malignant tumours were staged according to FIGO criteria. Of the ovarian cancer patients, 15 had stage I-II, 17 stage III and 12 stage IV. Twenty-seven primary ovarian tumours were histologically serous, seven were endometrial, three were mucinous and seven were undifferentiated. Twenty out of 43 patients (46%) with endometrial cancer were at stage I, 14 patients (32%) at stage II and nine patients (21%) at stage III and IV. Eighteen cervical cancer patients (27%) had stage I, 24 (36%) stage II and 24 (36%) stage III and IV. All endometrial cancers were adenocarcinomas, while among cervical tumours 57 were squamous and nine adenocarcinomas. All patients with breast cancer had stage I or stage II disease. Tumours were classified from G1 to G3 according to the degree of histological differentiation.

The first serum sample was always taken before therapy. A postoperative sample was usually obtained 4-6 weeks after surgery or just before the first chemotherapy cycle. Serial TAG 72 measurements were performed before each chemotherapy cycle and during follow-up.

Stability, progression and regression of disease were defined according to WHO criteria (1979).

Fifteen patients with primary ovarian cancer underwent a short course of rIFN alpha-2b (Intron, ESSEX, Milano, Italy). Patients received $3 \times 10^6$ U daily i.m. of rIFN alpha-2b for 3 consecutive days and TAG 72 levels were measured daily for 3 days after the first injection.

Venous blood samples for marker determinations were separated by centrifugation and aliquots were stored at $-20°C$ until assayed. TAG 72 assay was performed using two commercially available kits (Centocor CA 72.4 RIA, Sorin B 72.3 MKS IRMA). The Centocor CA 72.4 RIA is a standard solid phase radioimmunoassay which utilises two monoclonal antibodies, B 72.3 and cc 49, a second generation antibody with greater affinity to B 72.3 and similar specificity. In the B 72.3 MKS IRMA, B 72.3 is used both on the solid phase immunoabsorbent and as a radiolabelled probe to subsequently detect and quantify the bound antigen. Intra- and inter-assay variations were 8.1% and 11.9% for CA 72.4 RIA and 8.3% and 11.2% for B 72.3 MKS IRMA. The upper normal level was taken as 7 U $ml^{-1}$ and 5 U $ml^{-1}$ for CA 72.4 RIA and B 72.3 MKS IRMA, respectively. This level corresponded to the mean value + 2 s.d. of our control group. CA 125 and SCC were measured using commercially available kits from CIS, Compagnie ORIS Industrie, SA and Abbott Laboratories. The upper limits of normal were taken as 35 U $ml^{-1}$ and 2.5 ng $ml^{-1}$ for CA 125 and SCC respectively. Using these cut-off values, 93% and 94% of our healthy patients had normal marker values respectively.

Marker titres were defined as increasing or decreasing when changes were greater than 50% of the previous value. The $\chi^2$ test and Fisher's exact test were used to evaluate the distribution of marker values according to different variables.

### Results

### *Pre-treatment evalution*

Table I shows the median level, range and percentage of positivity of TAG 72 in patients with benign and malignant gynaecological tumours. Although CA 72.4 RIA assay seems to be more sensitive than B 72.3 MKS IRMA, no statistically significant difference between the two assays was found in any of the gynaecological malignancies examined. Moreover, linear regression analysis revealed a statistically significant correlation ($P<0.01$) between values obtained with the two assays (data not shown). Therefore, in continuing the study we only referred to data obtained with the CA 72.4 RIA assay.

Abnormally high TAG 72 values were seen most frequently in ovarian tumours. Elevated levels of TAG 72 occurred in 29 out of 44 patients (66%) with primary epithelial ovarian cancer. Among patients with uterine tumours, abnormal TAG 72 levels were only found in 15 (23%) and six (14%) cervical and endometrial cancer patients, respectively. None of the 20 patients with breast cancer had elevated CA 72.4 levels. All patients with endometriosis and endometrial hyperplasia showed TAG 72 levels within the normal range. Only one of 12 (8%) patients with benign ovarian tumours and one out of 15 (7%) with uterine fibromyomatosis had slightly increased TAG 72 levels.

Table II compares the distribution of CA 72.4 and CA 125 in ovarian cancer as related to stage, histology and tumour grade. TAG 72 positivity was not significantly related to stage, histotype or histological grading. Using a combination of TAG 72 and CA 125 assays, the overall sensitivity was only slightly increased, with 89% of the serum samples showing a positive reaction in at least one test compared to 86% for CA 125 alone. We compared the sensitivity of TAG 72 with that of SCC in patients with cervical cancer. As shown in Table III, the sensitivity of TAG 72 was significantly lower with respect to that of SCC. When a combination of two assays was used the overall sensitivity rose to 64% compared to 59% of the SCC alone.

**Table I** Serum levels of TAG 72 measured with CA 72-4 RIA and B 72-3 MKS IRMA in patients with malignant and benign gynaecological diseases and control subjects

| *Type of sera* | *No. of sera tested* | *CA 72-4 RIA >7 U ml⁻¹(%)* | *B 72-3 MKS IRMA >5 U ml⁻¹(%)* |
|---|---|---|---|
| Controls | 66 | 6% *(0;0–21) | 6% (0;0–6) |
| Ovarian cancer | 44 | 66% (7.7; 0–541) | 61% (8.8;0–121) |
| Cervical cancer | 66 | 23% (0; 0–32) | 17% (0;0–36) |
| Endometrial cancer | 43 | 14% (0; 0–13) | 12% (0;0–16) |
| Breast cancer | 20 | – | – |
| *Benign conditions* | | | |
| Ovarian tumours | 12 | 8% (0;0–11) | n.d. |
| Fibromyomatosis | 15 | 7% (0;0–9) | n.d. |
| Endometriosis | 20 | – | n.d. |
| Endom. hyperplasia | 10 | – | n.d. |

Median and range levels are indicated in parentheses.

**Table II** Serum positivity of TAG 72 and CA 125 in ovarian cancer patients according to stage, histology and tumour grade

| | *No. of sera tested* | *TAG 72 >7 U ml⁻¹ (%)* | *CA 125 >35 U ml⁻¹ (%)* | *Two assays combined[a] (%)* |
|---|---|---|---|---|
| *Stage* | | | | |
| I–II | 15 | 67 | 80 | 87 |
| III | 17 | 65 | 88 | 88 |
| IV | 12 | 66 | 92 | 92 |
| Total | 44 | 66 | 86 | 89 |
| *Histology* | | | | |
| Serous | 27 | 67 | 77 | 82 |
| Endometr. | 7 | 57 | 100 | 100 |
| Mucinous | 3 | 100 | 100 | 100 |
| Undiff. | 7 | 57 | 100 | 100 |
| *Grading* | | | | |
| G1 | 7 | 100 | 86 | 100 |
| G2 | 12 | 67 | 92 | 100 |
| G3 | 17 | 75 | 88 | 88 |

[a]At least one positive test.

### *Postoperative evaluation*

One month after completion of primary therapy all patients with no clinical evidence of disease had TAG 72 levels within the normal range.

In patients with advanced ovarian cancer who underwent cytoreductive surgery, a good correlation was found between TAG 72 levels and residual tumours after surgery (Table IV). Five out of 12 (42%) patients, with residual disease >0.5 cm showed abnormal TAG 72 serum levels with respect to one out of four patients (25%) without residual disease. However, CA 125 was more sensitive than TAG 72 in detecting residual disease after surgery.

### *TAG 72 and clinical course of disease*

In 18 patients TAG 72 levels were measured during chemotherapy, consisting of three courses of high dose cisplatin (200 mg $m^{-2}$) (Benedetti Panici *et al.*, 1987). Figure 1 shows serum TAG 72 levels before and at completion of chemo-

**Table III** Combined sensitivity of SCC with TAG 72 in 66 patients with cervical cancer

| | *No. patients* | *(%)* |
|---|---|---|
| SCC>2.5 ng ml⁻¹ | 39 | (59) |
| TAG 72>7 U ml⁻¹ | 15 | (23) |
| Two assays combined[a] | 42 | (64) |

[a]At least one positive test.

**Table IV** Correlation of TAG 72 serum levels with residual tumour after surgery in ovarian cancer patients

| *Residual tumour* | *No. of sera tested* | *TAG 72>7 U ml⁻¹* | | *CA 125>35 U ml⁻¹* | |
|---|---|---|---|---|---|
| | | *No.* | *(%)* | *No.* | *(%)* |
| No residual disease | 4 | 1 | (25) | 1 | (25) |
| Residual disease | | | | | |
| <0.5 | 3 | 0 | – | 1 | (33) |
| >0.5 | 12 | 5 | (42) | 11 | (92) |

**Figure 1** Serum TAG 72 levels before and after chemotherapy, according to clinical response. The dotted line shows the upper limit of normal (7 U $ml^{-1}$).

therapy in relation to clinical response to treatment. Ten out of 11 patients who responded to treatment had decreasing TAG 72 titers while five out of seven patients with stationary or progressive disease showed persistently elevated TAG 72 serum levels after chemotherapy.

In pre-treated ovarian cancer patients, TAG 72 levels correlated with disease status at follow-up. Seventy-five per cent of patients with recurrent disease had an abnormal TAG 72 value while only one out of 11 patients with no evidence of disease had a positive value (Table V).

In patients with positive TAG 72 serum assay, serial antigen measurements correlated well with the clinical course of the disease. However, in none of the 10 patients with high pre-treatment TAG 72 levels and in whom marker levels were serially measured during follow-up did changes in TAG 72 levels provide a better correlation with the eventual course of disease than changes in CA 125 levels (data not shown).

### *TAG 72 levels and findings on second-look*

After chemotherapy 19 patients with ovarian cancer underwent second-look laparoscopy (Table VI). TAG 72 serum levels were found to be within the normal range in all six patients with no histological or cytological evidence of disease, as well as in three patients with residual disease of less than 2 cm. Only five out of 10 patients with residual disease greater than 2 cm had high TAG 72 serum levels. Again, CA 125 proved to be a better indicator of persistent disease at second-look than TAG 72. Moreover, two out of three patients with positive CA 125 and no evidence of disease at second-look relapsed within 6 months after second-look.

### *Effect of rIFN alpha-2b on TAG 72 levels*

To test whether TAG 72 serum levels could be up-regulated by treatment with IFN (Greiner *et al.*, 1984; Guadagni *et al.*, 1986), 15 patients with primary or recurrent ovarian cancer received rIFN alpha-2b for 3 consecutive days ($3 \times 10^6$ U daily i.m.) and TAG 72 was measured daily for 3 days after the first injection. As shown in Table VII, rIFN alpha-2b did not significantly modify TAG 72 serum levels, irrespective of the antigen basal levels. In only one case did TAG 72 levels markedly decrease after IFN administration.

### Discussion

The need to improve the diagnosis and the clinical monitoring of gynaecological tumours has encouraged the measurement of tumour associated serum antigens. In this report we analysed the clinical usefulness of a novel human pancarcinoma antigen called TAG 72 in patients with gynaecological malignancies. Sixty-six per cent of patients with primary epithelial ovarian cancer showed abnormal concentrations of TAG 72 antigen, which were found to be correlated with the clinical course of disease during chemotherapy and follow-up. However, in our series, CA 125 proved to be significantly more sensitive than TAG 72 in detecting not only primary ovarian tumours but also residual disease after surgery or before second-look. Moreover, the combined evaluation of CA 72.4 and CA 125 does not result in increased sensitivity and improved clinical usefulness. Interestingly, however, normal TAG 72 levels have been found in patients with endometriosis, benign ovarian tumours and fibromyomatosis. This is relevant since high CA 125 levels have been found in patients with endometriosis and benign ovarian tumours (Kenemans *et al.*, 1988; Scambia *et al.*, 1988). Our results therefore are in agreement with previous histological findings showing a high specificity of TAG 72 for malignant tissues with respect to normal tissues and benign lesions. Thor *et al.* (1986) reported B 72.3 immunoreactivity in more than 70% of epithelial ovarian tumours, while normal ovarian tissues and 26 out of 27 various benign ovarian tumours were negative. Interestingly, the only benign tumours with TAG 72 expression showed an unusual glandular complexity.

**Table V** Correlation of TAG 72 serum levels with clinical status of disease at follow-up in ovarian cancer patients

| *Clinical status* | *No. of sera tested* | *TAG 72>7 U ml⁻¹* No. | (%) | *CA 125>35 U ml⁻¹* No. | (%) |
|---|---|---|---|---|---|
| Evidence of disease | 24 | 18 | (75) | 20 | (83) |
| No evidence of disease | 11 | 1 | (9) | 1 | (9) |

**Table VI** Correlation of TAG 72 and CA 125 serum levels with disease status at second-look in ovarian cancer patients

| *Disease status* | *No. of cases* | *TAG 72>7 U ml⁻¹* No. | (%) | *CA 125>35 U ml⁻¹* No. | (%) |
|---|---|---|---|---|---|
| No evidence of disease | 6 | 0 | – | 3 | (50) |
| Evidence of disease | | | | | |
| <2 cm | 3 | 0 | – | 1 | (33) |
| >2 cm | 10 | 5 | (50) | 6 | (60) |

**Table VII** Variation of TAG 72 during recombinant interferon alpha-2b administration

| *Case* | *Days* I | II | III | IV |
|---|---|---|---|---|
| 1 | 0 | 0 | 0 | 0 |
| 2 | 0 | 0 | 0 | 0 |
| 3 | 0 | 0 | 0 | 0 |
| 4 | 0 | 0 | 0 | 0 |
| 5 | 0 | 0 | 0 | 0 |
| 6 | 0 | 0 | 0 | 0 |
| 7 | 0 | 0 | 0 | 3 |
| 8 | 0 | 3 | 3 | 3 |
| 9 | 4 | 3 | 4 | 4 |
| 10 | 4 | 5 | 4 | 4 |
| 11 | 7 | 3 | 3 | 3 |
| 12 | 14 | 16 | 20 | 16 |
| 13 | 20 | 15 | 8 | 8 |
| 14 | 22 | 26.2 | 19 | 21 |
| 15 | 122 | 95 | 101 | 112 |

Recombinant alpha-2b interferon was administered ($5 \times 10^6$ U daily, i.m.) for 3 consecutive days (days I–III) and TAG 72 was measured daily for 3 days after the first injection.

Based on these findings it can be suggested that TAG 72 assay could be of some value in the differential diagnosis of pelvic tumours in order to reduce the false positive results of CA 125. Moreover, the high specificity of MAB B 72.3 for malignant tissues suggests that this antibody could be useful for tumour radioimmunodetection and possibly immunotherapy. It is also worth noting that Colcher *et al.* (1984) reported a prolonged binding of radiolabelled B 72.3 when used for the *in situ* radioimmunodetection of human colon carcinoma xenografts. Recently, preliminary data from Surwit *et al.* (1989) indicated that labelled B 72.3 could be successfully used for immunoscanning of patients with ovarian cancer. Compared to ovarian cancer in uterine tumours, the sensitivity of TAG 72 assay was very low: 14% in endometrial cancer and 23% in cervical cancer. In these neoplasias only patients with advanced disease were found to have significantly positive TAG 72 serum levels. Moreover, in cervical cancer quantification of TAG 72 does not improve the sensitivity of SCC alone.

As previously reported by Klug *et al.* (1986), elevated levels of TAG 72 are only rarely present in the sera of patients with breast cancer. This is rather surprising since MAB B 72.3 was generated using a membrane-enriched extract of a breast tumour metastasis as immunogen and has high level of reactivity with at least 50% of all breast carcinomas (Colcher *et al.*, 1981). Although the discrepancy between the tissue and serological expression might reflect the heterogeneity of antigen expression in malignant cells within a tumour (Stramignoni *et al.*, 1983), it can also be hypothesised that TAG 72 might be shed into circulation by different tumours at different rates. These findings also suggest that the serum TAG 72 assay cannot be used for screening candidates for tumour radioimmunodetection with radiolabelled B 72.3.

Because the sensitivity of radioimmunometric methods employing MoAb may be significantly influenced by the type of combination of reagent employed, we analysed our panel of sera using two different commercially available TAG 72 detection kits.

Our results did not show any significant advantage of TAG 72 measurement with a double antibody assay with respect to a single antibody assay, suggesting that at least in gynaecological malignancies this antigen contains multiple epitopes for B 72.3. Much interest has been focused on the finding that the surface expression of TAG 72 is increased by IFNs both *in vitro* on human breast and colon cancer cells (Grenier *et al.*, 1984; Guadagni *et al.*, 1987) and *in vivo* in human tumour xenograft in nude mice (Grenier *et al.*, 1986). However, the systemic administration of rIFN alpha-2b to 15 patients with ovarian cancer and different basal values of TAG 72 did not stimulate TAG 72 levels. Our negative findings can be explained as follows: (1) a more prolonged exposure to rIFN alpha-2b and/or a longer lead time after IFN administration could be necessary in order to observe an increase of TAG 72 levels; (2) different types of IFNs could be more effective than rIFN alpha-2b in the stimulation of TAG 72 expression; (3) IFNs could stimulate the surface expression of TAG 72 without a concomitant release of the antigen in the circulation. The first two possibilities seem rather unlikely. Several species of rIFN alpha, including rIFN alpha B, are able to stimulate the surface binding of MAB B 72.3 at very low concentrations with a lead time between rIFN treatment and TAG 72 increase of 24–36 h (Greiner *et al.*, 1986). It is also worth noting that using the same experimental procedure as that employed in this study we detected a marked increase of the circulating levels of a 90,000 Da antigen with a close temporal correlation between *in vitro* and *in vivo* data (Iacobelli *et al.*, 1988; Scambia *et al.*, 1990). Our data agree with the recent findings of Boyer *et al.* (1989), who were unable to demonstrate any IFN-induced increase of TAG 72 expression in six ovarian cancer cell lines *in vitro*.

In conclusion, our findings suggest that the serum assay of TAG 72 has only a limited value in the clinical management of patients with gynaecological malignancies. Further studies should be carried out to verify the possible role of B 72.3 in the pre-operative differential diagnosis of pelvic masses as well as in tumour radioimmunodetection.

### References

BAST, R.C., KLUG, T.L., ST. JOHN, E. *et al.* (1983). A radioimmunoassay using a monoclonal antibody to monitor the course of epithelial ovarian cancer. *N. Engl. J. Med.*, **9**, 883.

BENEDETTI PANICI, P., GREGGI, S., SCAMBIA, G. *et al.* (1987). High-dose (200 mg/m$^2$) Cisplatin-induced neurotoxicity in primary advanced ovarian cancer patients. *Cancer Treat. Rep.*, **7**, 6669.

BENEDETTI PANICI, P., SCAMBIA, G., BAIOCCHI. G. *et al.* (1989). Predictive value of multiple tumor marker assay at second look procedures in ovarian cancer. *Gynecol. Oncol.*, **35**, 286.

BEREK, J.S., KNAPP, R.C., MALKASIAN, G.D. *et al.* (1986). CA 125 serum levels correlated with second-look operations among ovarian cancer patients. *Obstet. Gynecol.*, **67**, 685.

BOYER, C.M., DAWSON, D.V., SHARON, E.V. *et al.* (1989). Differential induction of interferons of major histocompatibility complex-encoded and non-major histocompatibility complex-encoded antigens in human breast and ovarian carcinoma cell lines. *Cancer Res.*, **49**, 2928.

COLCHER, D., HORAN HAND, P., NUTI, M. *et al.* (1981). A spectrum of monoclonal antibodies reactive with human mammary tumor cells. *Proc. Natl Acad. Sci. USA*, **78**, 3199.

COLCHER, D., KEENAN, A.M., LARSON, S.M. *et al.* (1984). Prolonged binding of a radiolabeled monoclonal antibody (B72-3) used for the in situ radioimmunodetection of human colon carcinoma xenografts. *Cancer Res.*, **44**, 5744.

GREINER, J.W., HORAN HAND, P., NOGUCHI, P. *et al.* (1984). Enhanced expression of surface tumor-associated antigens on human breast and colon tumor cells after recombinant human leukocyte alpha interferon treatment. *Cancer Res.*, **44**, 3209.

GREINER, J.W., FISHER, P.B., PESTKA, S. *et al.* (1986). Differential effects of recombinant human leukocyte interferons on cell surface antigen expression. *Cancer Res.*, **46**, 4984.

GREINER, J.W., GUADAGNI, F., NOGUCHI, P. *et al.* (1986). Recombinant interferon enhances monoclonal antibody targetting of carcinoma lesions in vivo. *Science*, **235**, 895.

GUADAGNI, F., SCHLOM, J., JOHNSTON, W.W. *et al.* (1987). Recombinant human interferons mediate enhancement of tumor antigen expression on human tumor cells isolated from pleural effusion and ascites. *J. Interferon Res.*, **7**, 798.

IACOBELLI, S., SCAMBIA, G., NATOLI, C. *et al.* (1988). Recombinant human leukocyte interferon alpha 2b stimulates the synthesis and release of a 90K tumor-associated antigen in human breast cancer cells. *Int. J. Cancer*, **42**, 182.

JOHNSON, V.G., SCHLOM, J., PATERSON, A.J. *et al.* (1985). Analysis of a tumor-associated glycoprotein (TAG 72) identified by monoclonal antibody B72-3. *Cancer Res.*, **46**, 850.

KATO, H. & MORIOKA, T. (1977). Radioimmunoassay for tumor antigen of human cervical squamous cell carcinoma. *Cancer*, **40**, 1621.

KATO, H., TAMAI, K., MORIOKA, H. *et al.* (1984). Tumor antigen TA-4 in the detection of recurrence in cervical squamous cell carcinoma. *Cancer*, **54**, 1544.

KENEMANS, P., BAST, R.C., YEDEME, C.A. *et al.* (1988). CA 125 and polymorphic epithelial mucin as serum tumor markers. *Cancer Rev.*, **11/12**, 119.

KLUG, T.L., SATTLER, M.A., COLCHER, D. *et al.* (1986). Monoclonal antibody immunoradiometric assay for an antigenic determinant (CA 72) on a novel pancarcinoma antigen (TAG-72). *Int. J. Cancer*, **38**, 661.

MAURO, T., SHIBATA, K., KIMURA, A. *et al.* (1985). Tumor associated antigen, TA-4, in the monitoring of the effects of therapy for squamous cell carcinoma of the uterine cervix. *Cancer*, **56**, 302.

NILOFF, J.M., BAST, R.C., SCHAETZL, E.M. *et al.* (1985) Predictive value of CA 125 antigen levels in second-look procedures for ovarian cancer. *Am. J. Obstet. Gynecol.*, **151**, 981.

SCAMBIA, G., BENEDETTI PANICI, P., BAIOCCHI, G. *et al.* (1988). Measurement of a monoclonal-antibody-defined antigen (90K) in the sera of patients with ovarian cancer. *Anticancer Res.*, **8**, 761.

SCAMBIA, G., BENEDETTI PANICI, P., IACOBELLI, S. *et al.* (1990). Recombinant interferon alpha-2b enhances the circulating levels of a 90K tumor associated antigen in patients with gynecological and breast malignancies. *Cancer*, **65**, 1325.

SCHILTHUIS, M.S., ALDERS, J.G., BOUMA, J. *et al.* (1987). Serum CA 125 levels in epithelial ovarian cancer: relation with findings at second-look operations and their role in the detection of tumor recurrence. *Br. J. Obstet. Gynaecol.*, **94**, 202.

SENEKJIAN, E.K., YOUNG, J.M., WEISER, P.A. *et al.* (1987). An evaluation of squamous cell carcinoma antigen in patients with cervical squamous cell carcinoma. *Am. J. Obstet. Gynecol.*, **157**, 433.

STRAMIGNONI, D., BOWEN, R., ATKINSON, B. *et al.* (1983). Differential reactivity of monoclonal antibodies with human colon adenocarcinomas and adenomas. *Int. J. Cancer*, **31**, 543.

SURWIT, E., CHILDERS, J., GRAHAM, V. *et al.* (1989). Phase II clinical studies of monab B 72.3-GYK-DTPA. *Proc. Am. Soc. Clin. Oncol.*, **8**, 152.

THOR, A., OHUCHI, N., SZPAK, C.A. *et al.* (1986). Distribution of oncofetal antigen tumor-associated glycoprotein-72 defined by monoclonal antibody B72.3. *Cancer Res.*, **46**, 3118.

THOR, A., GORSTEIN, F., OHUCHI, N. *et al.* (1986). Tumor-associated glycoprotein (TAG 72) in ovarian carcinomas defined by monoclonal antibody B72.3. *J. Natl Cancer Inst.*, **76**, 995.

WORLD HEALTH ORGANIZATION (1979). *WHO Handbook for Reporting Results of Cancer Treatment*. WHO: Geneva.